# Development of a predictive model for vitamin D deficiency based on the vitamin D status in young Japanese women: A study protocol

**DOI:** 10.1371/journal.pone.0264943

**Published:** 2022-03-10

**Authors:** Akiko Kuwabara, Eiji Nakatani, Naoko Tsugawa, Hideaki Nakajima, Satoshi Sasaki, Kenichi Kohno, Kazuhiro Uenishi, Masaru Takenaka, Kyoko Takahashi, Akihiro Maeta, Nobuko Sera, Kaori Kaimoto, Masako Iwamoto, Hisaya Kawate, Mayumi Yoshida, Kiyoshi Tanaka

**Affiliations:** 1 Department of Clinical Nutrition, Graduate School of Comprehensive Rehabilitation, Osaka Prefecture University, Osaka, Japan; 2 Division of Medical Statistics, Graduate School of Public Health, Shizuoka Graduate University of Public Health, Shizuoka, Japan; 3 Department of Health and Nutrition, Osaka Shoin Women’s University, Osaka, Japan; 4 Earth System Division, National Institute for Environmental Studies, Ibaraki, Japan; 5 Department of Social and Preventive Epidemiology, School of Public Health, The University of Tokyo, Tokyo, Japan; 6 Division of Health Data Science, Translational Research Center for Medical Innovation, Kobe, Japan; 7 Division of Nutritional Physiology, Kagawa Nutrition University, Saitama, Japan; 8 Graduate School of Life Science, Kobe Women’s University, Hyogo, Japan; 9 Department of Food Science and Nutrition, School of Food Science and Nutrition, Mukogawa Women’s University, Hyogo, Japan; 10 Department of Nutrition Science, University of Nagasaki, Nagasaki, Japan; 11 Department of Human Life and Science, Kagoshima Women’s College, Kagoshima, Japan; 12 Department of Nutritional Sciences, Nakamura Gakuen University, Fukuoka, Japan; 13 Department of Nutrition, Tenshi College, Hokkaido, Japan; 14 Faculty of Nutrition, Kobe Gakuin University, Hyogo, Japan; Holbaek Sygehus, DENMARK

## Abstract

**Background:**

Vitamin D deficiency (VDD) is associated with an increased risk for lifestyle-related diseases. In Japan, VDD is quite prevalent in all age groups, with its high risk in young women. Furthermore, its association during pregnancy with gestational hypertension and low birth weight has also been reported. VDD can be diagnosed by serum 25-hydroxyvitamin D [25(OH)D] levels, which, however, is not suited for screening. Therefore, we will create a predictive model for serum 25(OH)D concentration and prevalence of VDD based on such data as region, sun exposure habit, and vitamin D intake in young women.

**Methods:**

From 2020 to 2022, we conduct a cross-sectional study of 600 young women in four regions of Japan, identify the indices associated with serum 25(OH)D concentrations such as sun exposure habits, habitual vitamin D intake, ultraviolet-B irradiation, seasons (summer and winter) and latitude, and construct prediction models for serum 25(OH)D concentrations and VDD risk. This study has been registered with UMIN-CTR (ID: UMIN000041527).

**Results:**

One hundred and fifteen subjects have been collected from 6 institutions in winter as of May 2021. When data from more than 200 subjects have become available, we will conduct the interim analysis, summarize the data by region and facility, review the inclusion criteria for analysis, and check for missing values and outliers. Prediction models for serum 25(OH)D concentration and VDD will be determined in the final analysis when all cases have been collected.

**Conclusions:**

A screening tool for VDD risk to be developed in our study based on the predictive model would help the public and medical professionals prevent lifestyle-related diseases through improving VDD. Additionally, the results may serve as the scientific basis for determining the appropriate vitamin D intake and sun exposure standards.

## Introduction

Vitamin D insufficiency/deficiency increases the risk of various diseases, such as osteoporotic fracture, infectious diseases, diabetes mellitus, cardiovascular diseases, and cancer, and their primary prevention through improving vitamin D status can play pivotal roles [1]. Although attention has been mainly paid to reducing the elderly musculoskeletal syndromes by vitamin D, it can also play important roles in the various aspects of health promotion in younger subjects [2–5].

Considering the high prevalence of vitamin D insufficiency/deficiency in almost all age groups in Japan [6–8], its improvement is of utmost importance for preventing the above-mentioned diseases. To develop a strategy to ameliorate vitamin D insufficiency/deficiency, it is required to detect the factors related to serum 25-hydroxyvitamin D [25(OH)D] concentration, which is the best indicator of vitamin D nutritional status.

Serum 25(OH)D concentrations reflect not only the vitamin D intake but also the production in the skin by UV irradiation [9]. Since Japan has marked seasonal variation in climate and regional divergence in latitudes, a study covering multiple seasons and residential latitudes is needed. However, there have been few reports of studies conducted in different residential latitudes in Japan, and they are limited to reports on children and pregnant women [10,11]. On the other hand, there are some studies in Europe demonstrating that calculated UV irradiation levels is an important predictor for serum 25(OH)D concentrations [12,13].

Maternal vitamin D status has been reported to be significantly associated with fetal and neonatal vitamin D status and can affect the outcomes during pregnancy and neonatal and infant development [14]. For example, vitamin D deficiency (VDD) during pregnancy was significantly associated with pre-eclampsia [15–17], gestational diabetes [16], preterm birth, and small-for-gestational-age (SGA) [18]. Therefore, normalization of serum 25(OH)D levels in women of childbearing possibility is very important for safe delivery and birth health.

Based on such background, the present study will collect data on the seasons, lifestyle, and dietary habits associated with serum 25(OH)D concentrations in 600 young women from four different latitudes to identify predictors of serum 25(OH)D concentrations and develop a predictive model for vitamin D deficiency.

## Materials and methods

### Study design and participants

**Fig 1:**
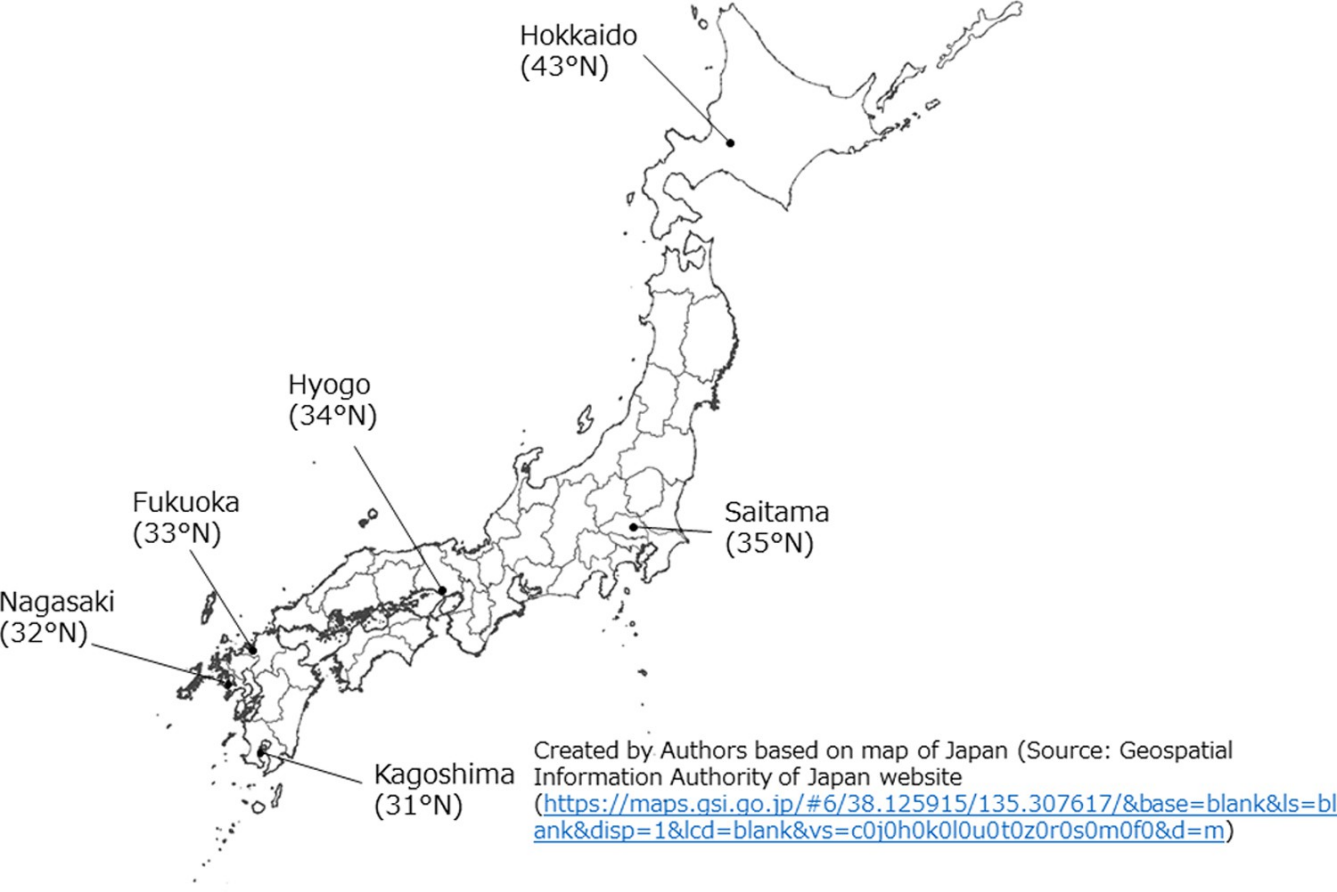
Prefectures and their latitudes included in this study. Prefectures included in the study are indicated by points. The figure created by Authors based on map of Japan (Source: Geospatial Information Authority of Japan website (https://maps.gsi.go.jp/#6/38.125915/135.307617/&base=blank&ls=blank&disp=1&lcd=blank&vs=c0j0h0k0l0u0t0z0r0s0m0f0&d=m).

This is a cross-sectional study. Because of the potential influence of latitude in predicting serum 25(OH)D concentrations, we plan to recruit 600 healthy women between the ages of 18 and 40 years living in Japan, who participate in the study from November 2020 to September 2022 from four regions: Hokkaido/Tohoku, Kanto, Chubu/Kinki/Shikoku, and Kyushu/Okinawa. The prefectures and their latitudes included in this study are shown in Fig 1.

Women aged 18 and 40 years at the study entry and who have given voluntary written consent to participate in this study are being enrolled. Participants with comorbidities such as chronic kidney disease, autoimmune disease, cancer, liver disease, diabetes mellitus, and those under sun exposure restriction due to such diseases as xeroderma pigmentosum are excluded. Those with chronic gastrointestinal diseases such as celiac disease, chronic pancreatitis, inflammatory bowel disease, and cystic fibrosis are also excluded considering its possible interference with vitamin D absorption.

This study is conducted in two seasons: summer (June to September) and winter (December to February), each corresponding to the highest and lowest serum 25(OH)D values, respectively [19,20].

**Fig 2:**
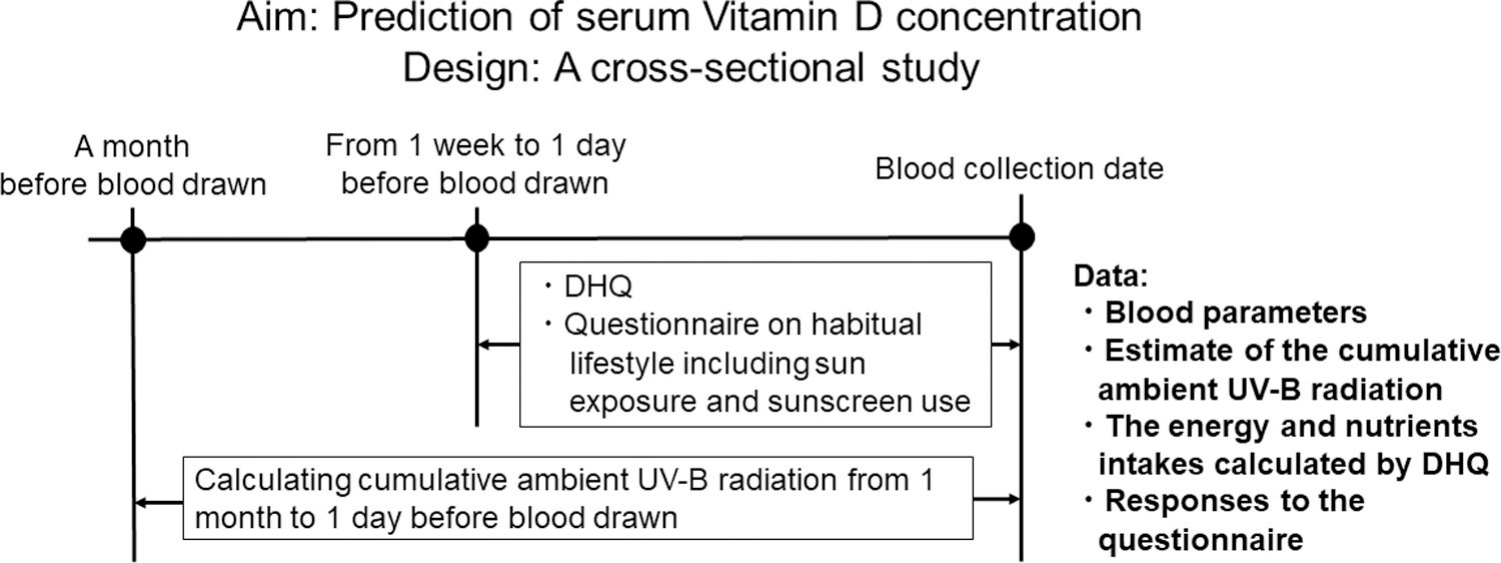
The study schedule in each season. A cross-sectional study is conducted for 600 young women in four regions (Hokkaido/Tohoku, Kanto, Chubu/Kinki/Shikoku, and Kyushu/Okinawa) of Japan from 2020 to 2022. This study is performed twice in the summer (June to September) and in the winter (December to February) in each region. The figure describes the study schedule in each season.

The participants are asked to fill out a questionnaire about their dietary history and habitual lifestyle during the week prior to their blood draw. Participants’ blood samples are collected in the morning and stored at 4 to 10°C in the refrigerator before centrifugation. Biochemical tests are performed on the next day, and then the residual serum samples are stored at -80°C for the measurement of serum 25(OH)D concentration. The study schedule is shown in Fig 2.

Both a comprehensive self-administered diet history questionnaire (DHQ) and the Lifestyle Questionnaire assess habitual food intake and sun exposure over the past month, based on that serum 25(OH)D concentrations reflect the vitamin D status for approximately one month.

### Ethical considerations and registration

This study conforms to the Ethical Principles for Medical Research Involving Human Subjects issued by the Ministry of Health, Labour and Welfare and the Ministry of Education, Culture, Sports, Science, and Technology in Japan. This research protocol has been approved by the ethics committees at institutions written below;

1. The committee on research ethics, The Graduate School of Comprehensive Rehabilitation Osaka Prefecture University, August 3, 2020 (the date of approval), 2020–302 (the approval number), 2. The ethical committee in Osaka Shoin Women’s University, Feb 10, 2021 (the date of approval), 20–15 (the approval number), 3. The research ethics review committee of Kagawa Nutrition University, November 18, 2020 (the date of approval), 308 (the approval number), 4. The ethics committee of human research of Kobe Women’s University, December 4, 2020 (the date of approval), 2020-17-2 (the approval number), 5. The research ethics committee of Mukogawa Women’s University, November 25, 2020 (the date of approval), 20–64 (the approval number), 6. The committee on research ethics, University of Nagasaki, October 7, 2020 (the date of approval), 439 (the approval number), 7. The committee on research ethics, Kagoshima Women’s College, October 1, 2020 (the date of approval), R2-1 (the approval number), 8. The human research ethics committee of Nakamura Gakuen University, December 4, 2020 (the date of approval), 20–012 (the approval number), 9. The ethics committee of Tenshi College, October 12, 2020 (the date of approval), 2020-21(the approval number).

This study has been registered with UMIN-CTR (ID: UMIN000041527).

### Planned size

The sample size required for this study was calculated to be 600 cases; based on a previous study showing that 52% of females aged 17–18 years had VDD (serum 25(OH)D level below 20 ng/mL) [8], and assuming that the proportion of VDDs is also 50% in the study population, we expect 300 individuals to have VDDs. Finally, we expect 30% of the subjects to drop out of the study due to incomplete data sets and withdrawal of consent.

### Dietary assessment

A dietary assessment is performed. Trained research staffs check the participants’ answers to a comprehensive self-administered diet history questionnaire (DHQ) and resolve the ambiguity. Vitamin D intake is calculated by the revised comprehensive self-administered DHQ, which is a semi-quantitative questionnaire assessing habitual dietary intake over the past month, and already validated in previous studies [21–23]. Vitamin D intake is energy intake- adjusted by the density method and expressed as an amount (μg) per 1000 kcal of daily energy intake [24]. Estimates of habitual daily intake of energy, nutrients, and foods are calculated based on the Standard Tables of Food Composition in Japan [25].

### The questionnaire on habitual lifestyle including sun exposure and sunscreen use

The questionnaire on habitual lifestyle is studied as in “Indices obtained from a questionnaire response” of Table 1 and S1 File. S1 File is used in the present study. Investigation items include characteristics of the subjects, habitual sun exposure, and habitual dietary intake. These items were adopted from the recently developed “Vitamin D Deficiency Questionnaire for Japanese: VDDQ-J” [20]. We have not performed a pre-test for the questionnaire employed in the current study. However, VDDQ-J, which constitutes the basis for the current questionnaire has been validated and already published [20]. The vitamin D abundant fish was defined as fish containing more than 10 μg of vitamin D in the habitual intake value of Japanese, such as salmon, sardine, saury, flounder, eel, herring, and grunt.

**Table 1:** Demographic characteristics and questionnaire response.

Items	Category to be counted (unit to be summarized)
**Indices to be measured or calculated**	
** 1. Biochemical indices** ** **Serum 25(OH)D	(ng/mL)
** **Serum intact PTH	(pg/mL)
** 2. UV-B exposure related index** ** **The UV-B irradiation	(mJ/cm^2^)
** 3. Energy and Nutrients intakes (**calculated from DHQ)	
** **Energy	(kcal/day)
** **Protein	(g/day)
** **Fat	(g/day)
** **Carbohydrates	(g/day)
** **Calcium	(mg/day)
** **Calcium adjusted for energy intake	(mg/1000 kcal)
** **Vitamin D	(μg/day)
** **Vitamin D adjusted for energy intake	(μg/1000 kcal)
**Indices obtained from a questionnaire response** ** 1. Characteristics of the Subject** ** **Age	(y)
** **The residential area	
	Hokkaido Tohoku
	Kanto
	Chubu
	Kinki
	Chugoku, Shikoku
	Kyushu and Okinawa
** **The season of blood draw	Summer (July to September)
	Winter (December to February)
** **The Fitzpatrick skin type	I: Pale white skin, blue/green eyes, blond/red hair (Always burns, does not tan)
	II: Fair skin, blue eyes (Burns easily, tans poorly)
	III: Darker white skin (Tans after initial burn)
	IV: Light brown skin (Burns minimally, tans easily)
	V: Brown skin (Rarely burns, tans darkly easily)
	VI: Dark brown or black skin (Never burns, always tans darkly)
** **BMI (kg/m^2^)	<25 kg/m^2^
	≥25 kg/m^2^
** **Medical history	Hypertension
	Dyslipidemia
	Diabetes mellitus
	Liver diseases
	Renal diseases
	Thyroid disease
	Anemia
	Allergic disease
** **Medication	Yes
	No
** **Supplement use	No
	Vitamin D
	Calcium
	B vitamins
	Vitamin C
	Multivitamin
	Iron
	Others
** **Smoking status	Never smoked
	Formerly smoked
	Currently smoking
** **Alcohol consumption	Never
	Less than once per month
	2 to 3 times per month
	2 to 3 times per week
	More than 4 times per week
** **Sleeping hours	Over 9 hours
	8 hours
	7 hours
	6 hours
	Less than 5 hours
** **Exercise habit	More than twice per week
	Once per week
	1 to 2 times per month
	Rarely
**2. Habitual sun exposure** ** **Suntan within the past 12 months	Yes
	No
** **Sun exposure in the last 3 months	Always
	More often than not
	Sometimes
	Infrequently
	Never
** **Time regularly spent outside on weekdays (min) * Even outside, time spent in the trains or cars is not included because of the UV-B’s limited penetration of glass.	7 to 8 AM
	8 to 9 AM
	9 to 10 AM
	10 to 11 AM
	11 to 12 AM
	12 AM to 1 PM
	1 to 2 PM
	2 to 3 PM
	3 to 4 PM
	4 to 5 PM
	5 to 6 PM
** **Time spent outside on weekdays	≥3 h/d
	2 to <3 h/d
	1 to <2 h/d
	<1h/d
	rarely
** **Time regularly spent outside on weekends (min) * Even outside, time spent in the trains or cars is not included because of the UV-B’s limited penetration of glass.	7 to 8 AM
	8 to 9 AM
	9 to 10 AM
	10 to 11 AM
	11 to 12 AM
	12 AM to 1 PM
	1 to 2 PM
	2 to 3 PM
	3 to 4 PM
	4 to 5 PM
	5 to 6 PM
** **Time spent outside on weekends	≥3 h/d
	2 to <3 h/d
	1 to <2 h/d
	<1h/d
	rarely
** **Sunscreen use	Never (less than 1 day a week)
	Rarely (1 or 2 days a week)
	Sometimes (3 or 4 days a week)
	More often than not (5 or 6 days a week)
	Always (everyday)
** **Sunscreen use for arms and legs	No
	Yes
** **Paying attention to UV exposure	No
	Yes
** **Recently wearing clothes	Arms and legs exposed
	Without skin exposed
**3. Habitual dietary intake** ** **Habitual intake of fish	More than 4 days a week
	2 to 3 days a week
	1 to 2 days a week
	Less than 1 day a week
** **Habitual intake of the vitamin D abundant fish (e.g. salmon, sardine, saury, flounder, eel, herring and grunt)	More than 4 days a week
	2 to 3 days a week
	1 to 2 days a week
	Less than 1 day a week

### Assay of serum 25(OH)D concentration

Serum vitamin D metabolites are measured by liquid chromatography tandem mass spectrometry (LC-APCI-MS/MS) as previously described with some modification [26], i.e. derivation of extracted vitamin D metabolites with by 4-[2-(6,7-dimethoxy-4-methyl-3-oxo-3,4-dihydroquinoxalyl) ethyl]-1,2,4-triazoline-3,5-dione (DMEQ-TAD) in order to obtain the high sensitivity by increasing ionization efficiency [27]. Coefficient of variation (CV) for intra- and inter-assay were 3.4−9.2% and 11.9% for 25(OH)D, and 13.1−19.3% and 14.7% for 24,25(OH)_2_D, respectively. Total serum 25(OH)D level is calculated by their summation. VDD refers to 25(OH)D level less than 20 ng/mL. Serum intact parathyroid hormone (iPTH) is measured by electro chemiluminescnce immunoassay at SRL, Inc. (Tokyo, Japan).

### Estimation of the cumulative ambient UV-B irradiation

We calculate the ultraviolet-B (UV-B) radiation flux density as detailed below, using a radiative-transfer code called ‘SMARTS2 (Simple Model of the Atmospheric Radiative Transfer of Sunshine, version 2)’ developed by Gueymard to calculate the flux density of solar radiation on the ground surface *E(λ)* for wavelength region between 280 and 4,000 nm [28], based on which we estimate the cumulative ambient UV-B irradiation from 1 month to 1day before blood drawn.

In particular, for wavelengths from 280 to 1,700 nm, calculations can be made at 1 nm intervals. UV spectrum of wavelength *λ* at the horizontal plane surface is expressed as follows;

$$E(\lambda )=E_{bn}(\lambda )\;\cos\;\theta_{z}+I_{d}(\lambda )$$

where

*E* (*λ*): UV flux density of the wavelength, *λ*,

*E*_*bn*_ (*λ*): UV spectrum of the direct component of solar radiation,

*θ*_*z*_: solar zenith angle,

*I*_*d*_ (*λ*): UV spectrum of the diffusive component of solar radiation.

This equation allows us to calculate the UV spectrum *E*_*bn*_ (*λ*) and *I*_*d*_ (*λ*) at the ground surface for a given season, time, and place under a cloudless sky. We apply this calculation to the sites where subjects are recruited at each participating university or college in the study. The UV flux reaching the top of the atmosphere from the sun is corrected for the distance between the earth and the sun for each day of the year. Total ozone at each site is given by measurements of NASA’s OMI [29] and OMPS [30] satellites which have been reporting the ozone total column data on a regular basis. The optical thickness of aerosols is observed under a cloudless sky at the Tsukuba station, Japan Meteorological Agency, using a sun-photometer that split the direct solar radiation into 368, 500, 675, 778, and 862 nm portions, which yield the Angstrom parameters, *α* and *β*. The data for aerosols for Tsukuba is used when there are no data available for aerosols at study sites. Rayleigh scattering is related to the number of air molecules that may generate atmospheric pressure. UV spectra have been observed by the Japan Meteorological Agency, every hour on a daily basis with Brewer spectra available for every 0.5 nm interval [31].

### Statistical analysis

The continuous variable will be expressed as mean ± standard deviation or median (interquartile range), and a categorical variable as frequency (percentage). Mann-Whitney U test and Kruskal-Wallis test will be used for the comparison between two groups and more than two groups, respectively. The chi-squared test will be used for categorical data.

An interim summary will be performed when the number of registered cases exceeds 200. The data will be summarized by region and institution. The inclusion criteria for analysis will be reviewed. We will also check for missing values and outliers. Predictors and prediction models will be determined in the final analysis when all cases have been collected.

Multivariate linear and logistic regression models are used to determine the predictors and predictive models for serum 25(OH)D concentration and VDD as outcomes, respectively. The regression coefficients and odds ratios will be calculated, and their 95% confidence intervals and p-values by Wald test will be calculated. Candidate predictors include age, residential area, skin type, smoking status, medication history, habitual drinking, physical activity, the season of blood collection, habitual sun exposure time and frequency, sunscreen use, the experience of suntan within the past 12 months, vitamin D supplement use, the habitual avoidance of sun exposure with parasol use or wearing long sleeve clothes, weekly frequency of vitamin D abundant fish consumption, and cumulative ambient UV-B irradiation.

Two-thirds of the randomly selected data will be used to build the predictive model, and the remaining data will be used to validate the built model. In the test set, variables shown to be significant in the univariate analysis and previously reported predictors are inserted into the multivariate model to construct the predictive model. In the validation set, the predictive performance of the prediction model is checked with the degrees of freedom-adjusted R-squared or AIC for linear regression analysis, and with the c-index for logistic regression analysis. In addition, as model calibration, the predicted and observed values are compared using Cook’s D for linear regression analysis and Hosmer-Lemeshow test for logistic regression analysis. Reporting on the construction of the predictive model will be done following the TRIPOD statement [32].

Statistical significance will be set at a two-tailed p-value < 0.05 for all analyses. All statistical analyses will be performed according to the statistical analysis plan decided before the analysis.

## Results and discussion

We plan to register 150 cases each in the summer and winter quarters of 2021–2022. Case registration has been delayed due to the spread of coronaviruses, and 115 cases were collected from 6 institutions by March 2021. We plan to conduct an interim analysis when the number of registered cases reaches 200 or more, and we plan to complete case registration and conduct the final analysis by around September 2022.

The purpose of this study is to determine how serum 25(OH)D concentration in young women is affected by such factors as latitude, season, diet, and lifestyle, and build a prediction model to predict serum 25(OH)D concentrations. The results of this study may provide a scientific basis for the forthcoming Dietary Reference Intakes and other various guidelines. We also plan to develop a tool to screen the risk of VDD and make it available through our website so that individuals can monitor their vitamin D status and easily assess their risk of vitamin D deficiency or insufficiency, which will lead to the prevention of lifestyle-related diseases by improving VDD.

This study is characterized by collecting data on UV-B exposure and lifestyle habits from four different latitudes (seven sites). Although previous reports are available in Japan on the circulating 25(OH)D levels at different latitudes [10,11], they suffer from the narrow range of latitudes [11], and lack of data on UV-B exposure. Another previous study conducted in Hokkaido (43°N) and Kumamoto (33°N) revealed a strong association between latitude and lifestyle [19]. Against this background, we will clarify how latitude and lifestyle affect UV exposure in this study.

There have been some previous papers reporting serum 25(OH)D concentrations in summer and winter, which has yielded conflicting results such as a significant correlation of serum 25(OH)D with UV exposure time and UV energy only in summer [19,20], and significant prediction of 25(OH)D level by UV index but with opposite direction of the association according to the blood collection period [12]. Then, the present study including the determination of serum 25(OH)D concentrations both in summer and winter at different UV irradiance levels together with various demographic variables would be of help clarifying the relationship between ambient UV-B irradiation and serum 25(OH)D levels on a seasonal basis.

A previous study has reported that the use of sunscreens and sun protection tools did not affect serum 25(OH)D concentrations in multivariate regression models [19]. This paper, however, included both sexes, which can be a confounding variable, and reports on the effect of the use of sunscreens and sun protection tools on serum 25(OH)D concentrations are quite limited in young women except in pregnant women [11]. Then, the study design of the present study including only women can rather be a strength, and suited for clarifying the effect of use of sunscreen and sun protection tools reduce serum 25(OH)D concentrations.

Individual UV irradiation dose can be evaluated either by direct measurement or estimation. As the former, the use of wearable devices is useful enabling a more accurate assessment of individual UV doses, which, however, is a burden to the participants. Then, we have not adopted such devices, and estimate the UV exposure for each region. The association of estimated UV irradiation dose and serum 25(OH)D levels has previously been reported [12,13]. For example, Cardoso et al [12] used the UV index obtained from the Portuguese Institute of Oceanography and Atmospheric Research. O’Sullivan et al. estimated the cumulative and weighted daily ambient dose of UV-B at wavelengths where vitamin D synthesis is possible [13].

Much more strong association between the dietary vitamin D intake and serum 25(OH)D levels could be expected than that in European and American studies, with the possible bases described below. Previous European reports on the ultraviolet effects on vitamin D status had a methodological flaw; no data on dietary vitamin D intake [12,13]. Another important issue to be considered is the markedly different prevalence of subjects under vitamin D supplementation between Europe and Japan. It was as high as 49.9% in the European study [13], whereas it was only 5.1% in a Japanese study for pregnant women with an age distribution similar to that in our study [11]. In the above-mentioned European studies, it is quite likely that the overwhelming effects of supplementation have obscured the contribution of dietary vitamin D. In Japan with a much lower percentage of vitamin D supplementation, a strong association between dietary vitamin D intake and serum vitamin D concentration could be expected, unlike the results from European and American studies.

There are some obvious limitations in this study. First, since the study subjects are limited to young women, caution is needed in the generalization of the current results. Second, all participants are undergraduate nutrition students, likely with a higher level of knowledge about nutrition than the general population, leading to more accurate responses to the DHQ. Hence, the possibility of selection bias cannot be ruled out. Third, since this is a cross-sectional study, a causal relationship between serum 25(OH)D level and the predictors cannot be definitely concluded.

## Conclusions

In this study, we will investigate various indices related to VDD in young Japanese women, construct a model for predicting VDD, and develop a screening tool to determine the risk of VDD, which will be useful for disease prevention through the improvement of VDD.

## Supporting information

S1 File A questionnaire on vitamin D status related indices. A questionnaire used in the present study (in Japanese) was described. The English translation is also shown.
(DOCX)
