# Supplementary material for: Development of a predictive model for vitamin D deficiency based on the vitamin D status in young Japanese women: A study protocol
Source: PLoS One. 2022 Mar 10;17(3):e0264943. doi: 10.1371/journal.pone.0264943 (PMC8912175; doi:10.1371/journal.pone.0264943)
Supplement: S1 File — A questionnaire used in the present study (in Japanese) was described. The English translation is also shown. (DOCX) [file pone.0264943.s001.docx]

**A questionnaire on vitamin D status related indices**

| (1) Area of residence | 1. Hokkaido 2. Tohoku 3. Kanto 4. Chubu 5. Kinki 6. Chugoku, Shikoku 7. Kyushu and Okinawa |
| --- | --- |
| (2) The season of blood draw | 1. Summer (July to September) 2. Winter (December to February) |
| (3) Age(y) | (y) |
| (4) Height (cm) | (cm) |
| (5) Body weight | (kg) |
| (6) Smoking status | 1．Never smoked 2．Formerly smoked 3．Currently smoking |

If your answer is 1 or 2, please fill in the table below.

|  | Cumulative number of cigarettes smoked | Number of smoking years |
| --- | --- | --- |
| Formerly smoked | cigarettes | y |
| Currently smoking | cigarettes | y |

| (7) Alcohol consumption | 1．Never 2．Less than once per month3．2 to 3 times per month4．2 to 3 times per week5．More than 4 times per week |
| --- | --- |
| (8) Medications taken habitually | 1．Yes 2．No |
| If your answer is 1, please indicate the specific product name. | ( ) |
| (9) Supplements taken habitually | 1. Vitamin D 2. Calcium 3. B vitamins 4. Vitamin C 5. Multivitamin 6. Iron 7. Others |
| Please indicate the specific product name of the supplement you are taking. | ( ) |
| How much vitamin D does your supplement contain? | 1. Vitamin D dosage per day  （Vit. D: 　　　　　　 μg/day） 2．Vitamin D content in the supplement is unknown.  3. The supplement you are taking does not contain vitamin D. |

| (10) Past or current medical history | |  |  |  |
| --- | --- | --- | --- | --- |
|  | Past illnesses（Please circle the applicable one） | Current illnesses（Please circle the applicable one） | Age of onset | Current medication for each disease |
| **１．Hypertension** | Yes　・　No | Yes　・　No | y | Yes　・　No |
| **２．Dyslipidemia** | Yes　・　No | Yes　・　No | y | Yes　・　No |
| **３．Diabetes mellitus** | Yes　・　No | Yes　・　No | y | Yes　・　No |
| **４．Hyperuricemia** | Yes　・　No | Yes　・　No | y | Yes　・　No |
| **５．Arrhythmia** | Yes　・　No | Yes　・　No | y | Yes　・　No |
| **６．Cerebral hemorrhage** | Yes　・　No | Yes　・　No | y | Yes　・　No |
| **７．Heart diseases** | Yes　・　No | Yes　・　No | y | Yes　・　No |
| **８．Cancer** | Yes　・　No | Yes　・　No | y | Yes　・　No |
| **９．Thyroid disease** | Yes　・　No | Yes　・　No | y | Yes　・　No |
| **10．Liver diseases** | Yes　・　No | Yes　・　No | y | Yes　・　No |
| **11．Pancreatic disease** | Yes　・　No | Yes　・　No | y | Yes　・　No |
| **12．Gastric and duodenal ulcers** | Yes　・　No | Yes　・　No | y | Yes　・　No |
| **13．Chronic kidney disease** | Yes　・　No | Yes　・　No | y | Yes　・　No |
| **14．Renal diseases other than Chronic kidney disease** | Yes　・　No | Yes　・　No | y | Yes　・　No |
| **15．Anemia** | Yes　・　No | Yes　・　No | y | Yes　・　No |
| **16．Hypotension** | Yes　・　No | Yes　・　No | y | Yes　・　No |
| **17．Allergic disease** | Yes　・　No | Yes　・　No | y | Yes　・　No |
| **18．Osteoporosis** | Yes　・　No | Yes　・　No | y | Yes　・　No |
| **19．Depression** | Yes　・　No | Yes　・　No | y | Yes　・　No |
| **20．Others indicate the specific name of disease** | Yes　・　No | Yes　・　No | y | Yes　・　No |
|  | Yes　・　No | Yes　・　No |  | Yes　・　No |
|  | Yes　・　No | Yes　・　No |  | Yes　・　No |

| (11) Sleeping hours | 1. Over 9 hours 2. 8 hours 3. 7 hours 4. 6 hours 5. Less than 5 hours |
| --- | --- |
| (12) Exercise habit | 1．More than twice per week 2．Once per week  3．1 to 2 times per month 4．Never |
| (13) Have you received a suntan in the past 12 months? | 1．Yes 2．No |
| (14) In the last 3 months, how often were you in the sunlight while lightly dressed | 1．Always 2．More often than not  3．Sometimes 4．Infrequently 5．Never |

(15) Time regularly spent outside on weekdays (min)

|  | 0 min | 1～< 10 min | 10～< 20 min | 20～< 30 min | 30～< 40 min | 40～< 50 min | 50～< 60 min |
| --- | --- | --- | --- | --- | --- | --- | --- |
| 7 to 8 AM |  |  |  |  |  |  |  |
| 8 to 9 AM |  |  |  |  |  |  |  |
| 9 to 10 AM |  |  |  |  |  |  |  |
| 10 to 11 AM |  |  |  |  |  |  |  |
| 11 to 12 AM |  |  |  |  |  |  |  |
| 12 AM to 1 PM |  |  |  |  |  |  |  |
| 1 to 2 PM |  |  |  |  |  |  |  |
| 2 to 3 PM |  |  |  |  |  |  |  |
| 3 to 4 PM |  |  |  |  |  |  |  |
| 4 to 5 PM |  |  |  |  |  |  |  |
| 5 to 6 PM |  |  |  |  |  |  |  |

* Even outside, time spent in the trains or cars is not included because of the UV-B’s limited penetration of glass.

| (16) How much time did you spend outdoors during the daytime on weekdays during the past month? | 1．>3 hours per day  2．2 to <3 hours per day 3．1 to <2 hours per day 4．< 1hour per day  5．Rarely |
| --- | --- |

(17) Time regularly spent outside on weekends (min)

|  | 0 min | 1～< 10 min | 10～< 20 min | 20～< 30 min | 30～< 40 min | 40～< 50 min | 50～< 60 min |
| --- | --- | --- | --- | --- | --- | --- | --- |
| 7 to 8 AM |  |  |  |  |  |  |  |
| 8 to 9 AM |  |  |  |  |  |  |  |
| 9 to 10 AM |  |  |  |  |  |  |  |
| 10 to 11 AM |  |  |  |  |  |  |  |
| 11 to 12 AM |  |  |  |  |  |  |  |
| 12 AM to 1 PM |  |  |  |  |  |  |  |
| 1 to 2 PM |  |  |  |  |  |  |  |
| 2 to 3 PM |  |  |  |  |  |  |  |
| 3 to 4 PM |  |  |  |  |  |  |  |
| 4 to 5 PM |  |  |  |  |  |  |  |
| 5 to 6 PM |  |  |  |  |  |  |  |

* Even outside, time spent in the trains or cars is not included because of the UV-B’s limited penetration of glass.

| (18) How much time did you spend outdoors during the daytime on weekends during the past month? | 1．>3 hours per day  2．2 to <3 hours per day 3．1 to <2 hours per day 4．< 1hour per day  5．Rarely |
| --- | --- |

| (19) How often do you apply sunscreen when going outside? | 1．Always 2．More often than not  3．Sometimes 4．Infrequently 5．Never |
| --- | --- |
| (20) Do you use sunscreen on your arms and legs? | 1．Yes 2．No |
| (21) When you go out, do you usually walk in the shade, use a parasol, or take other actions to avoid UV rays? | 1．Yes 2．No |
| (22) Recently wearing clothes | 1. Arms and legs exposed 2. Without skin exposed |
| (23) The Fitzpatrick skin type | 1. Pale white skin, blue/green eyes, blond/red hair (Always burns, does not tan) 2. Fair skin, blue eyes (Burns easily, tans poorly) 3. Darker white skin (Tans after initial burn) 4. Light brown skin (Burns minimally, tans easily) 5. Brown skin (Rarely burns, tans darkly easily) 6. Dark brown or black skin (Never burns, always tans darkly) |
| (24) Habitual dietary intake Habitual intake of fish | 1. More than 4 days a week 2. 2 to 3 days a week 3. 1 to 2 days a week 4. Less than 1 day a week |
| (25) Habitual intake of the vitamin D abundant fish (e.g. salmon, sardine, saury, flounder, eel, herring and grunt) | 1. More than 4 days a week 2. 2 to 3 days a week 3. 1 to 2 days a week 4. Less than 1 day a week |

「日本人若年女性における血中ビタミンD濃度の実態調査及び

ビタミンD欠乏判定のための予測モデル開発研究のための横断的研究」

研究調査票

**記入日　２０　　年　　月　　日**

**ID（　　　　 　　　　　）**

**※調査票は両面印刷です。裏面も必ず回答いただくようお願い致します（表紙を含めて10ページです）。**

**記入日**　２０　　年　　月　　日 ID（　　　　 　　　　　）

　以下の質問について、該当する記号に〇を付け、自由記述については記入欄に回答してください。

**（1）居住地域を教えて下さい。**

| 1．北海道 | 2．東北 |
| --- | --- |
| 3．関東 | 4．中部 |
| 5．近畿 | 6．中国・四国 |
| 7．九州・沖縄 |  |

**（２）今の季節を回答して下さい。**

1．夏（7月～9月）

2．冬（12月～ 2月）

**（３）あなたの現在の年齢を教えてください。**

　　　　　　歳

**（４）あなたの身長を教えてください。**

　　　　　　cm

**（５）あなたの体重を教えてください。**

　　　　　　kg

**（６）喫煙状況をお聞きします。**

１．喫煙している

２．かつて喫煙していた

３．喫煙したことがない

**１又は２の場合は以下の表中に回答してください。**

|  | 喫煙状況（本数） | 喫煙状況（年数） |
| --- | --- | --- |
| かつて喫煙していた | （かつて）  本 | （かつて）  年 |
| 喫煙している | 本 | 年 |

**（７）飲酒状況について教えてください。**

1．週4回以上

2．週2～3回

3．月2～3回、週1回

4．月1回以下

5．全く飲まない

**（８）日常的に飲んでいるお薬はありますか。**

1．服用あり

2．服用なし

**1 =服用ありの場合、具体的な製品名を教えてください。（複数回答可）**

**製品名：**

**（９）日常的に飲んでいるサプリメントはありますか。（複数回答可）**

1．服用していない

2．ビタミンD

3．カルシウム

4．ビタミンB

5．ビタミンC

6．マルチビタミン

7．鉄剤

8．その他

**2～8．の方について、具体的な製品名を教えてください。複数の場合は下記を参照に全てお答え下さい。**

**例(３．カルシウムと５．ビタミンCを服用の場合:　3. A社 〇▼□、5. B社 ◆〇△●)**

**製品名：**

**使用されているサプリメント中のビタミンD服用量についておたずねします。**

※複数のサプリメントを使用されている場合には、合算値（1日あたり）を書いてください。

１．サプリメント中のビタミンD含有量が分かる（含有量から服用量を計算ください）

1日あたりビタミンD服用量　**µg**

２．サプリメント中のビタミンD含有量が不明（ビタミンDが含まれているか分からない、ビタミンD含有の記載があるが含有量は記載なしなど）

３．服用しているサプリメントにビタミンDは含まれていない

**（10）これまであるいは現在の病気の状況について表中に回答してください。**

20．その他　の場合には、具体的な病名を書いてください。

|  | 過去にかかった  （有または無に〇をしてください） | 現在かかっている（有または無に〇をしてください） | かかった年齢  （数字を書いてください） | 現在の治療薬の使用有無（有または無に〇をしてください） |
| --- | --- | --- | --- | --- |
| **１．高血圧症** | 有　・　無 | 有　・　無 | 歳 | 有　・　無 |
| **２．脂質異常症** | 有　・　無 | 有　・　無 | 歳 | 有　・　無 |
| **３．糖尿病** | 有　・　無 | 有　・　無 | 歳 | 有　・　無 |
| **４．高尿酸血症** | 有　・　無 | 有　・　無 | 歳 | 有　・　無 |
| **５．不整脈** | 有　・　無 | 有　・　無 | 歳 | 有　・　無 |
| **６．脳卒中（脳出血・脳梗塞等）** | 有　・　無 | 有　・　無 | 歳 | 有　・　無 |
| **７．心臓病（狭心症・心筋梗塞等）** | 有　・　無 | 有　・　無 | 歳 | 有　・　無 |
| **８．がん** | 有　・　無 | 有　・　無 | 歳 | 有　・　無 |
| **９．甲状腺疾患（バセドウ病、橋本病など）** | 有　・　無 | 有　・　無 | 歳 | 有　・　無 |
| **10．肝臓疾患** | 有　・　無 | 有　・　無 | 歳 | 有　・　無 |
| **11．膵臓疾患** | 有　・　無 | 有　・　無 | 歳 | 有　・　無 |
| **12．胃・十二指腸潰瘍** | 有　・　無 | 有　・　無 | 歳 | 有　・　無 |
| **13．腎不全** | 有　・　無 | 有　・　無 | 歳 | 有　・　無 |
| **14．腎不全以外の腎疾患** | 有　・　無 | 有　・　無 | 歳 | 有　・　無 |
| **15．貧血** | 有　・　無 | 有　・　無 | 歳 | 有　・　無 |
| **16．低血圧症** | 有　・　無 | 有　・　無 | 歳 | 有　・　無 |
| **17．アレルギー疾患** | 有　・　無 | 有　・　無 | 歳 | 有　・　無 |
| **18．骨粗しょう症** | 有　・　無 | 有　・　無 | 歳 | 有　・　無 |
| **19．うつ病** | 有　・　無 | 有　・　無 | 歳 | 有　・　無 |
| **20．その他**  **※具体的な病名を書いてください**  **病名：** | 有　・　無 | 有　・　無 | 歳 | 有　・　無 |

**（11） 平均睡眠時間を教えてください。**

1．9時間以上

2．8時間

3．7時間

4．6時間

5．5時間以下

**（12）運動（スポーツ）をどれくらいしますか。**

1．週2回以上

2．週1回

3．月1～2回

4．全くしない

**（13）この12ヶ月間で日焼けしたことはありましたか。（日焼けとは、肌が黒くなることを指します）**

1．はい

2．いいえ

**（14）この3ヶ月間に、軽装で日光を浴びたことはどれくらいありますか。（軽装とは、腕や足などが出るような服装を指します）**

1．いつも（毎日）

2．たいてい（5、6日/週）

3．ときどき（3、4日/週）

4．まれに（1、2日/週）

5．全くない（1日未満/週）

**※普段の日照時間・戸外活動時間について（15）（16）では平日、（17）（18）では休日について伺います。**

**（15）普段の平日（週の大半を占める日）の主な外出時間（屋外）についてお尋ねします。各時間帯の外出時間のうち、当てはまるところに〇をつけてください。**

**注意！　※電車や車内（ガラス越し）で過ごす時間は含みません。**

|  | 0分 | 1～10分未満 | 10～20分未満 | 20～30分未満 | 30～40分未満 | 40～50分未満 | 50～60分 |
| --- | --- | --- | --- | --- | --- | --- | --- |
| 7～8時 |  |  |  |  |  |  |  |
| 8～9時 |  |  |  |  |  |  |  |
| 9～10時 |  |  |  |  |  |  |  |
| 10～11時 |  |  |  |  |  |  |  |
| 11～12時 |  |  |  |  |  |  |  |
| 12～13時 |  |  |  |  |  |  |  |
| 13～14時 |  |  |  |  |  |  |  |
| 14～15時 |  |  |  |  |  |  |  |
| 15～16時 |  |  |  |  |  |  |  |
| 16～17時 |  |  |  |  |  |  |  |
| 17～18時 |  |  |  |  |  |  |  |

**（16）この1か月間の平日における、日中の1日あたりの戸外活動時間を教えてください。**

1．3時間以上

2．2時間以上、3時間未満

3．1時間以上、2 時間未満

4．1時間未満

5．ほとんど外に出なかった。

**（17）普段の休日の主な外出時間（屋外）についてお尋ねします。各時間帯の外出時間のうち、当てはまるところに〇をつけてください。**

**注意！　※電車や車内（ガラス越し）で過ごす時間は含みません。**

|  | 0分 | 1～10分未満 | 10～20分未満 | 20～30分未満 | 30～40分未満 | 40～50分未満 | 50～60分 |
| --- | --- | --- | --- | --- | --- | --- | --- |
| 7～8時 |  |  |  |  |  |  |  |
| 8～9時 |  |  |  |  |  |  |  |
| 9～10時 |  |  |  |  |  |  |  |
| 10～11時 |  |  |  |  |  |  |  |
| 11～12時 |  |  |  |  |  |  |  |
| 12～13時 |  |  |  |  |  |  |  |
| 13～14時 |  |  |  |  |  |  |  |
| 14～15時 |  |  |  |  |  |  |  |
| 15～16時 |  |  |  |  |  |  |  |
| 16～17時 |  |  |  |  |  |  |  |
| 17～18時 |  |  |  |  |  |  |  |

**（18）この1か月間の休日における、日中の1日あたりの戸外活動時間を教えてください。**

1．3時間以上

2．2時間以上、3時間未満

3．1時間以上、2 時間未満

4．1時間未満

5．ほとんど外に出なかった。

**（19）外出時にどれくらい日焼け止めを使いますか。**

1．いつも（毎日）

2．たいてい（5、6日/週）

3．ときどき（3、4日/週）

4．まれに（1、2日/週）

5．全くない（1日未満/週）

**（20）手脚に日焼け止めを塗りますか。**

１．はい

２．いいえ

**（21）普段外出する際、日陰を歩いたり日傘をさすなど、紫外線を避ける行動を意識していますか。**

1．はい

2．いいえ

**（22）ここ最近の服装に当てはまるものに〇をつけてください。**

1．半袖など、手足の露出がある

2．長袖で肌の露出がほとんどない

**（23）あなたの紫外線に対する肌の反応について当てはまるものに〇をつけてください。**

**※日焼け止めを使用しない状態の肌の反応（最近、日焼け止めを使用し続けている場合には、過去の状態を思い出して選択してください）**

1．常に赤くなり、決して皮膚色が濃くならない（肌の色：白）

2．常に赤くなり、その後少し皮膚色が濃くなる（肌の色：白）

3．時々赤くなり、必ず皮膚色が濃くなる（肌の色：白）

4．決して赤くならず、必ず皮膚色が濃くなる（肌の色：淡褐色）

5．元の皮膚色が濃い（肌の色：褐色）

6．元の皮膚色がとても濃い（肌の色：暗褐色や黒色）

**（24）魚の摂取状況についてお教えください。**

1．週4日以上

2．週2日以上4日未満

3．週1日以上2日未満

4．週1日未満

**（25）以下のグループに含まれる魚を週に何回摂取しているか教えてください。**

**さけ いわし　　さんま**

**かれい うなぎ 　 にしん**

**いさき かわはぎ**

1．週4日以上

2．週2日以上4日未満

3．週1日以上2日未満

4．週1日未満

**質問は以上です。お疲れ様でした。**
